# *s-dePooler*: determination of polymorphism carriers from overlapping DNA pools

**DOI:** 10.1186/s12859-019-2616-9

**Published:** 2019-01-22

**Authors:** Aleksandr Igorevich Zhernakov, Alexey Mikhailovich Afonin, Natalia Dmitrievna Gavriliuk, Olga Mikhailovna Moiseeva, Vladimir Aleksandrovich Zhukov

**Affiliations:** 1 Research Department of Non-Coronary Heart Diseases, Almazov National Medical Research Center, Ministry of Health of Russia, 2 Akkuratova St., St. Petersburg, 197341 Russia; 2 All-Russia Research Institute for Agricultural Microbiology (ARRIAM), 3 Podbelsky Ch., St. Petersburg - Pushkin, 196608 Russia

**Keywords:** Sample pooling, DNA pools, Overlapping pools, Depooling, Polymorphism discovery

## Abstract

**Background:**

Samples pooling is a method widely used in studies to reduce costs and labour. DNA sample pooling combined with massive parallel sequencing is a powerful tool for discovering DNA variants (polymorphisms) in large analysing populations, which is the base of such research fields as Genome-Wide Association Studies, evolutionary and population studies, etc. Usage of overlapping pools where each sample is present in multiple pools can enhance the accuracy of polymorphism detection and allow identifying carriers of rare-variants. Surprisingly there is a lack of tools for result interpretation and carrier identification, i.e. for “depooling”.

**Results:**

Here we present ***s-dePooler***, the application for analysis of pooling experiments data. ***s-dePooler*** uses the variants information (VCF-file) and the pooling scheme to produce a list of candidate carriers for each polymorphism. We incorporated ***s-dePooler*** into a pipeline (***dePoP)*** for automation of pooling analysis. The performance of the pipeline was tested on a synthetic dataset built using the 1000 Genomes Project data, resulting in the successful identification 97% of carriers of polymorphisms present in fewer than ~ 10% of carriers.

**Conclusions:**

***s-dePooler*** along with ***dePoP*** can be used to identify carriers of polymorphisms in overlapping pools, and is compatible with any pooling scheme with equivalent molar ratios of pooled samples. ***s-dePooler*** and ***dePoP*** with usage instructions and test data are freely available at https://github.com/lab9arriam/depop.

**Electronic supplementary material:**

The online version of this article (10.1186/s12859-019-2616-9) contains supplementary material, which is available to authorized users.

## Background

The investigation of DNA polymorphisms is a cornerstone of modern genetics research. Next generation sequencing (NGS) is widely used for the determination of polymorphisms and estimation of their frequency, yet the cost of large-scale DNA sequencing remains prohibitively high. Sample pooling (i.e., mixing DNA samples into pools for library preparation) substantially reduces the costs of such projects.

Dividing specimens into multiple pools yields additional benefits over using a single pool containing all the specimens. The smaller the pool, the lower is the required sequencing depth per specimen [1]. Furthermore, multiple pools can be used for the correction of sequencing errors [2]. Another strategy for enhancing the accuracy of polymorphism detection is to use overlapping pools, a strategy in which each sample is added to multiple pools. Overlapping pools make it possible to identify carriers of polymorphisms; thus, this strategy is extremely valuable for some applications, such as clinical trials.

Carrier identification is possible only if for each pair of analysed specimens, there is at least one pool containing only one of them (‘separating pool’) [3]. Several types of pooling scheme satisfying this requirement have been developed. One type of such pooling scheme is multi-dimensional pooling strategy [4] in which samples are allocated in N-dimensions space and are pooled for each “coordinate” in each axis. The approximate number of pools (k) can be estimated as $u\approx N\ast \sqrt[N]{n}$, where n – is the number of specimens and N – is the number of dimensions. Another pooling scheme is “logarithmic signature” [5] for which the minimal required number of pools is *k* ≈ *log*_2_ *n* + 1 [3].

Both pooling strategies above need pools of great size up to a half of the analysing population, resulting in the need for deep sequencing coverage. Partially this can be amended by using the “transversal shift” scheme. This strategy is focused on two main parameters: minimization of co-occurrence between specimens and construction of pools of constantly sized intersections [6, 7].

The success of allele carrier identification depends on the frequency of an allele in the population. If the analysed population contains more than one specimen carrying the minor allele, unambiguous identification might become impossible, since any pooling strategy inevitably leads to information loss. The larger the analysed population and the longer the analysed region, the more labour-intensive and computationally demanding identification of the carriers becomes, exacerbated by the possibility of there being multiple carriers of each polymorphism.

We developed ***s-dePooler***, an application for the determination of minor allele carriers (depooling) using the results of NGS sequencing of overlapping pools, the first application developed for this specific purpose. ***s-dePooler*** (*i*) estimates the most probable numbers of the minor allele copies in each pool (*Allele–Pool* distribution) based on the numbers of reference and alternative reads and (*ii*) determines subsets of specimens carrying that minor allele (*Allele–Sample* distribution) that satisfy the pooling scheme. We incorporated ***s-dePooler*** into ***dePoP*** (depooling pipeline) that we developed, which fully automates the analysis of large-scale pooling experiments.

### Modelling

The proportion of DNA strands containing a minor allele in a pool (*θ*) can be determined primarily by the ratio of minor allele copies (*m*) to all the allele copies in the pool (*k*). We assume *θ* as a beta-distributed random variable with parameters *M* and *R*:

$$ \theta \sim Beta\left(M,R\right), $$

where *M* = *m* ∙ *α*, *R* = (*k* − *m*) ∙ *α*, (*α* is the mixing precision coefficient, *α* ≥ 1).

Beta distribution is derived from the Dirichlet distribution used for the modelling of pool mixing. A pool comprising DNA of *k*/p samples of a *p*-ploid organism contains k individual allele copies. If equimolar sample mixing is assumed, the fraction of each individual allele in the pool can be modelled by the Dirichlet distribution of order *k* with equal-in-value parameters *α*_*i*_ = *α*.

The number of observed minor allele reads (*r*_*m*_) is assumed to be a binomial random variable with parameters *η* (the probability of getting a minor read) and *r*_*t*_ (the total number of reads mapped to the locus):

$$ {r}_m\sim Bin\left({r}_t,\eta \right). $$

The probability of getting a minor read (*η*) is connected to the proportion of minor allele DNA strands in a pool (*θ*) through correction by sequencing error rate (*Es*):

$$ \eta =\theta \bullet \left(1- Es\right)+\left(1-\theta \right)\bullet \frac{Es}{3}. $$

We define the discrete distribution (*Allele–Pool* distribution) specifying the probability of getting *r*_*m*_ minor allele reads among *r*_*t*_ given the numbers of minor allele copies in a pool (*m*) using a Bayesian approach. The probability mass function value at each *m* is determined as:

$$ P\left({r}_m|{r}_t,m\right)=\left\{\begin{array}{c}P\left({r}_m|{r}_t,\frac{Es}{3}\right),m=0\\ {}{\int}_0^1P\left({r}_m|{r}_t,\upeta \left(\theta \right)\right)\bullet P\left(\theta |\mathrm{M},\mathrm{R}\right) d\theta,\ 0<m<k\\ {}\ P\left({r}_m|{r}_t,1- Es\right),\ m=k\ \end{array}\right. $$

### Implementation

#### S-dePooler

Using the result of SNP-calling, namely the values of the AD vector (designated in VCF –specification, https://samtools.github.io/hts-specs/VCFv4.2.pdf) that represents the numbers of reads of the reported genetic variants, and *Allele–Pool* distribution, ***s-dePooler*** for each pool calculates the confidence interval with a given confidence level that most probably contains the minor allele copy number. Analysing the different combinations of the minor allele numbers in all pools, from the most likely combinations to the least likely, varying the numbers inside confidence intervals, ***s-dePooler*** defines the variants of the *Allele-Sample* distribution that could give a certain combination according to the pooling scheme.

To calculate the *Allele-Sample* distribution satisfying a certain combination of minor allele copy numbers in all pools, ***s-dePooler*** uses brute force search. It recursively permutes minor alleles distribution between specimens within a pool, pool by pool keeping only the distributions that satisfy the pre-set combination of allele copy numbers in all pools and the results of the function on higher levels, thereby cutting off erroneous branches after the first inconsistency. The recursion depth is equal to the number of pools (*u*). So the running time for each SNV lies within a factor $O\left(\prod \limits_i^u{C}_{m_i}^{2\bullet {n}_i}\right)$, where *n*_*i*_ – is the number of specimens in *i*-th pool, *m*_*i*_ – is the number of minor allele copies in *i*-th pool. Parallel execution is implemented. Since each variant of *Allele-Sample* distribution can be processed independently, as can each SNV, the algorithm should scale linearly with the increase of execution threads.

The computational complexity increases with the $\sum \limits_i^u{m}_i$ and achieves maximum when 50% of the alleles are “minor”. In order to keep the computational time reasonable, the program can be set to limit execution by time or by the number of steps. Although more efficient algorithms can be developed for specific pooling schemes this algorithm was developed specifically for use with any pooling scheme.

One of the coefficients present in the *Allele-Pool* distribution formula is α - the mixing precision coefficient. We propose two approaches allowing estimation of α in practice. The first is the estimation of instrument errors (pipettes, DNA concentration measuring devices, etc.) and the following calculation of possible variations of specimen percentages in mixed pools. The second approach is possible if tested specimens were already partly genotyped. Comparison of observed fractions of SNVs in known region with the expected values will make it possible to estimate the accuracy of specimen mixing. This task should be solved individually for any particular case based among other things on the purposes of the study as higher α value could lead to the increase of false-negative results, whereas underestimation of α could lead to false-positive results and/or enlargement of candidate lists.

#### dePoP

***dePoP*** is a pipeline designed for the automation of depooling process starting from raw NGS data. ***dePoP*** automatizes de-multiplexing, trimming, mapping of NGS-reads, SNP-calling of resulting mapped alignment and finally depooling of discovered genetic variants with ***s-dePooler***. The pipeline requires Perl (v.5) and the Bowtie2, Cutadapt and samtools tools to be accessible in the Perl-script runtime environment. Default SNP-caller integrated into the pipeline is Genome Analysis Toolkit (GATK) but it should be compatible with any SNP-caller that outputs VCF-files with AD-vector. ***s-dePooler*** and ***dePoP*** along with usage instructions and test data are available at https://github.com/lab9arriam/depop [8].

## Results

To test the application, Illumina reads of 104 individuals belonging to chromosome 11 of the human genome were downloaded from the 1000 Genomes project website [9]. A total of 24 pools were formed according to the ‘transversal shift’ scheme, with 13 samples per pool, so that each sample was present in three pools (see table in Additional file 1: Table S1). Reads were assigned randomly to each pool, so that for each of the 12 emulated pools, the number of reads was approximately 300 million (the expected number of high-quality reads from one lane of Illumina HiSeq 2000). To emulate the mixing of sequencing libraries and the sample pooling, the proportions of pool reads in an emulated lane were generated according to the Dirichlet distribution with all parameters equalling 4; proportions of sample reads in each pool were generated according to the Dirichlet distribution with all parameters equalling 3 (see table in Additional file 2: Table S2).

A region 17,463-bp long with a combined average coverage of more than 7739 for all libraries was selected for the testing of ***s-dePooler***. SNP-calling for each specimen was performed using Samtools [10]; the results were used to benchmark ***s-dePooler***. SNP calling of emulated pools was performed using GATK [11] and the resulting VCF file was analysed by ***s-dePooler*** (see table in Additional file 3: Table S3). Individual SNP calling detected 79 distinct polymorphic sites among 104 specimens in the investigated region. For 77 polymorphic sites present in fewer than ~ 10% of specimens (a threshold chosen for rare allele variants), ***s-dePooler*** successfully predicted all of the carriers 68 times, one or two carriers were missed for five sites, one site was not identified, and for the remaining three sites the *Allele–Sample* distribution was not calculated in the set time. The total run time for this with 6 threads was 22 m 16 s. Run time for the same task with 50 treads was 4 min 4 s, 5.5 times faster.

**Fig. 1:**
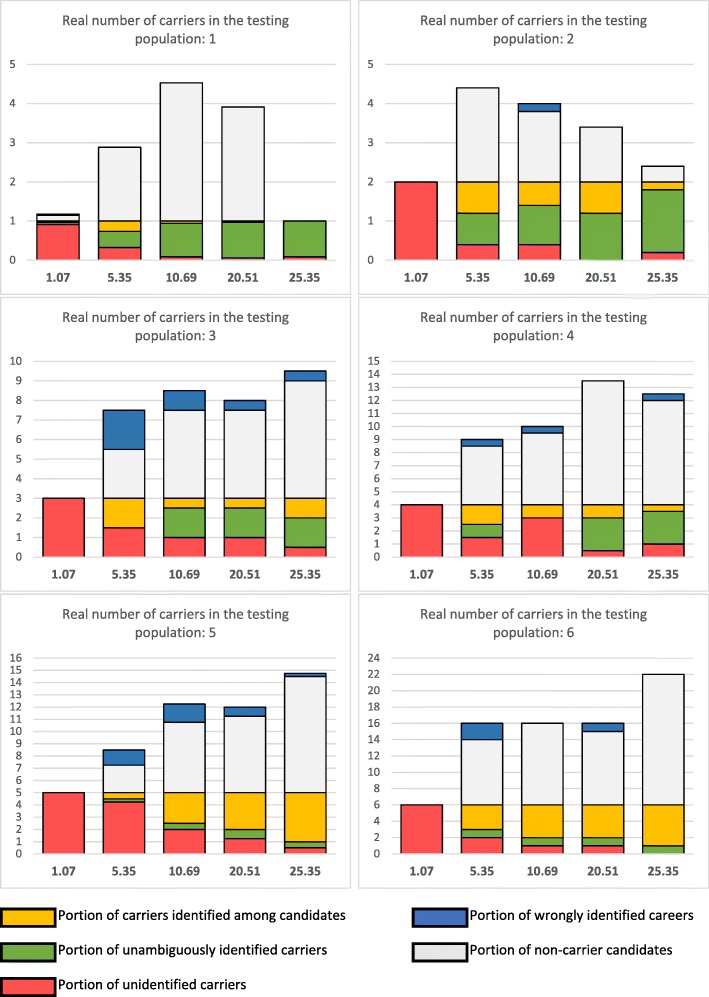
Average numbers of candidate carriers identified at different coverage. Legend: The average number of candidate carriers was calculated for all the SNVs in the analyzed region. Green color signifies the number of confidently identified carriers, yellow – the number of carriers among candidates, white – the number of false candidates, blue – the number of wrongly identified carriers, red – the number of unidentified carriers. The y axis represents the number of candidates for each SNV. Columns represent different coverages tested; the coverages (per pool, per specimen) are displayed under the axis

In order to determine the influence of sequencing depth on the tool we decided extract subsets of reads from the analyzed region. Since the dataset presented highly uneven coverage, we decided to filter out all the SNVs with coverage less than 200x for the lowest coverage. Resulting coverages were in range between 14x per single pool (approximately 1x coverage per specimen in a pool) to 329x per single pool (approximately 25x per specimen), the latter using all the available reads from the test dataset. The results of the tests are presented in Fig. 1 and Additional file 3: Table S3). The coverage affects the accuracy of the carrier detection, but we did observe that coverage of about 10x per specimen in a pool (third column from the left) was sufficient to confidently determine 85% of SNVs with a single carrier (91% for 25x coverage) and 50% of SNVs with two (80% for 25x coverage).

## Discussion

The test dataset example shows applicability of the chosen pooling scheme for a case when the polymorphic site is present in fewer than ~ 10% of specimens. If the frequency of a minor allele is higher than 10% another pooling scheme including greater number of pools and/or more frequent appearance of specimens in pool (e.g. each specimen in four different pools) might be more useful. We showed, that for our test data, that average coverage of about 10x allows to determine 85% of carriers of unique SNVs. This shows a possible application of such pooling schemes in TILLING (i.e., screening of mutant collections), since the majority of important SNVs in these specimens should be unique. This pooling strategy reduces the costs of library preparation by four and may allow easier sample multiplexing, an important factor to consider, since single lane output for Illumina platforms has increased in recent years.

The question of choosing the optimal pooling scheme should be considered for any particular case taking into account background knowledge on the object and expected genetic variability. Although the pooling scheme used in this study can easily be scaled for a bigger analysing population, the scaling leads to increment of pool sizes, which will require an exponential increase of sequencing depth in order to maintain the same confidence level. ***s-dePooler*** requires about 200 Mb of RAM per single thread, but the computational resources needed to solve an *Allele-Pool* distribution combination depends on the number of minor allele copies in the analysed population. So a single carrier may be quickly found in a pool set compiled from a thousand specimens, but if a half the analysed samples carry a “minor” allele, the computational time becomes unreasonable, even if the number of specimens is relatively small.

## Conclusions

***s-dePooler*** is the first program developed for the depooling of highly complex experiments. ***s-dePooler*** can identify carriers of polymorphisms in pools of large sizes and potentially in across a whole eukaryotic genome, a task not achievable with manual labour. ***s-dePooler*** requires (*i*) the pooling scheme to be in the form of a tsv-formatted file and (*ii*) a VCF file with an AD field for each pool (https://samtools.github.io/hts-specs/VCFv4.2.pdf). ***s-dePooler*** was incorporated into a Perl-based pipeline ***dePoP*** that we developed for the automation of depooling. Both tools are compatible with any pooling scheme in which specimens are pooled in equimolar ratios. This pipeline performs all of the necessary steps (read transformation, mapping and SNP calling) in a streamlined fashion. ***s-dePooler*** and ***dePoP*** along with usage instructions and test data are available at https://github.com/lab9arriam/depop [8].

## Availability and requirements

**Project name**: ***s-dePooler***, ***dePoP.***

**Project home page**: https://github.com/lab9arriam/depop

**Operating system(s)**: Platform independent.

**Programming language**: ***s-dePooler*** – Java; ***dePoP*** – Perl.

**Other requirements**: Java Runtime Environment 8 or higher, Perl 5 (for ***dePoP*** only).

**License**: GNU GPL version 3.

**Any restrictions to use by non-academics**: None.

## Additional files

Additional file 1: **Table S1.** Pooling scheme. (XLSX 16 kb)

Additional file 2: **Table S2.** Pooling emulation. (XLSX 16 kb)

Additional file 3: **Table S3.** Specimens carrying SNPs and results of depooling. (DOCX 16 kb)

## Abbreviations

AD: Allelic depth
NGS: Next generation sequencing
SNP: Single nucleotide polymorphism
SNV: Single nucleotide variant
VCF: Variant call format
