# Supplementary material for: s-dePooler: determination of polymorphism carriers from overlapping DNA pools
Source: BMC Bioinformatics. 2019 Jan 22;20:45. doi: 10.1186/s12859-019-2616-9 (PMC6343301; doi:10.1186/s12859-019-2616-9)
Supplement: Supplementary file 3 — Table S3. Specimens carrying SNPs and results of depooling. (DOCX 16 kb) [file 12859_2019_2616_MOESM3_ESM.docx]

**Additional file 3 Table S3: Specimens carrying SNPs and results of depooling.**

Specimens marked green were unambiguously identified by ***s-dePooler***.

Specimens marked yellow were identified as candidates along with non-carriers.

Specimens marked red remained unidentified.

Non-marked specimens were falsely identified as carriers.

| **Polymorphism** | **Carriers identified by individual SNP-calling** | **Carriers identified by *s-dePooler*** | |
| --- | --- | --- | --- |
|  |  | **Unambiguous** | **Candidates** |
| 11:62284468[A->C] | HG00651, HG00654, HG00334, HG01366, HG01790, HG01108 | - | NA18611, NA18603, HG01790, NA18572, HG00651, HG01125, HG01137, HG00699, HG00534, HG01375, HG00654, HG00334, HG01366, HG00449, HG00702, HG00613, HG00657, HG00689, HG01503, HG01108, HG00705, HG01506 |
| 11:62284797[C->T] | HG00653 | HG00653 | - |
| 11:62284845[C->A] | HG00501 | HG00501 | - |
| 11:62284888[G->A] | HG00321, HG01366, HG01174, HG01107, HG01171 | HG01171 | HG00321, HG00111, HG00671, HG01149, HG00334, HG01366, HG01174, HG01107 |
| 11:62285068[G->C] | HG01174 | HG01174 | - |
| 11:62285441[A->G] | HG00651, HG00654, HG00334, HG01366, HG01790 | - | NA18603, HG01173, HG01790, NA18572, HG00651, HG01137, HG00534, HG00699, HG01375, HG00654, HG00334, HG01366, HG00702, HG00613, HG00657, HG00689, HG01503, HG00705 |
| 11:62285571[C->T] | HG00232, NA06985, HG00328 | HG00232, NA06985, HG00328 | HG01507, HG00707, HG00690 |
| 11:62285665[C->T] | HG00651, HG00654, HG00334, HG01366, HG01790, HG01108 | - | NA18603, HG00651, HG01125, HG00534, HG00699, HG00654, HG00334, HG01366, HG00613, HG00657, HG01503, HG01108, HG01506, NA18611, HG01173, HG01790, NA18572, HG00581, HG01137, HG00342, HG01375, HG00702, HG00449, HG00689, HG00705 |
| 11:62285721[G->T] | HG00463, HG00693, NA18603, HG00708, HG00699, HG00702, HG00657, HG00701, HG00689, HG00619, HG00308 | - | NA18603, HG00651, HG01500, HG01149, HG00693, HG00699, HG00534, HG00698, HG00654, HG00334, HG00614, HG00657, HG00613, HG00619, HG01668, HG01503, HG01107, HG01108, HG01506, NA18611, NA18537, NA18572, HG01170, HG00321, HG00683, HG01137, HG01374, HG01375, NA18609, HG00328, HG00449, HG00689, HG00607, HG00708, HG01125, HG00672, HG00671, HG01124, HG01790, HG01350, HG01791, HG01670, HG00581, HG00580, HG01390, HG01510, HG00464, HG00463, HG00584, HG00342, HG01512, HG00583, HG00263, HG01353, HG01354, HG00702, HG00701, HG00308 |
| 11:62285847[G->A] | HG00308 | HG00308 | - |
| 11:62286607[C->G] | HG01374 | HG01374 | - |
| 11:62287828[T->C] | HG00651, HG00654, HG00334, HG01366, HG01790, HG01108 | - | NA18611, NA18603, HG01173, HG01790, NA18572, HG01125, HG00651, HG00342, HG00534, HG01375, HG00654, HG00328, HG00702, HG00613, HG00657, HG00689, HG01503, HG01108, HG00705, HG01506 |
| 11:62288130[G->A] | HG00650 | HG00650 | - |
| 11:62288649[G->C] | NA18611, NA18603, HG00708, NA18558, HG00464, HG00662, HG00650, HG00589, HG00699, HG00500, HG00449, HG00614, HG00613, HG00657, HG00704 | - | - |
| 11:62288666[G->A] | NA18558 | NA18558 | - |
| 11:62288851[C->T] | NA18609 | NA18609 | - |
| 11:62289508[C->T] | HG01108 | HG01108 | - |
| 11:62289739[A->T] | HG00607 | HG00607 | - |
| 11:62289932[C->T] | HG00533 | HG00533 | - |
| 11:62290719[A->G] | HG00651, HG00654, HG00334, HG01366, HG01790, HG01108 | - | NA18611, NA18572, HG00581, HG00651, HG01125, HG01137, HG00342, HG00699, HG00654, HG01375, HG00334, HG01366, HG00702, HG00449, HG00689, HG01108, HG00705, HG01506 |
| 11:62290876[G->A] | HG01500, HG01513, HG01375, HG01354, HG01124, HG01507, HG01670 | - | NA18611, HG01140, HG01350, HG01670, HG01390, HG00380, HG00464, HG01500, HG01512, HG00583, HG01513, HG01375, HG01672, HG01354, HG00334, HG01124, HG01507, HG00614, HG00449, HG00656, HG00689, HG00619, HG01108 |
| 11:62290938[C->G] | HG00672, HG00608 | HG00608 | HG00672, HG01669 |
| 11:62290979[T->C] | HG00463, HG00693, NA18603, HG00708, HG00699, HG00702, HG00657, HG00701, HG00689, HG00308 | - | - |
| 11:62291129[T->C] | HG01108 | HG01108 | - |
| 11:62291300[T->C] | HG01174 | HG01174 | - |
| 11:62291585[A->G] | HG01108 | HG01108 | - |
| 11:62291687[G->C] | HG00583 | HG00583 | - |
| 11:62291756[A->G] | HG00671, HG00705, HG00704 | HG00704 | HG01148, HG00671, NA18563, HG01174, HG01668, HG00705 |
| 11:62291953[A->T] | HG01149, HG00263, NA11881, HG01133, HG01375, HG00334, HG00308, HG00380 | - | - |
| 11:62292349[G->A] | HG01108 | HG01108 | - |
| 11:62292543[C->A] | HG01389 | HG01389 | - |
| 11:62292863[G->A] | HG00449 | HG00449 | - |
| 11:62292882[G->T] | all | all | - |
| 11:62292980[A->C] | HG01108 | HG01108 | - |
| 11:62293472[C->T] | HG01108 | HG01108 | - |
| 11:62293822[C->T] | 27 carriers, not listed | - | - |
| 11:62293913[T->G] | HG01350, HG00607 | HG01350, HG00607 | - |
| 11:62293916[C->A] | HG01108 | HG01108 | - |
| 11:62293948[G->A] | 68 carriers, not listed | - | - |
| 11:62294309[C->T] | HG01500, HG01504 | HG01500, HG01504 | - |
| 11:62294377[T->G] | HG01354 | HG01354 | - |
| 11:62294512[A->G] | HG01108 | HG01108 | - |
| 11:62294773[A->G] | HG01173 | HG01173 | - |
| 11:62294833[T->G] | HG01670 | HG01670 | - |
| 11:62294991[A->G] | HG00663, NA18572, HG00580 | HG00663, NA18572, HG00580 | HG00693, HG00328 |
| 11:62295076[G->A] | HG00449 | HG00449 | - |
| 11:62295149[T->G] | HG01365, HG01390 | HG01365, HG01390 | - |
| 11:62295361[C->A] | HG00651, HG00654, HG00334, HG01366, HG01790 | - | NA18603, HG01173, HG01790, NA18572, HG00651, HG00534, HG01375, HG00654, HG00702, HG00613, HG00657, HG00689, HG00705 |
| 11:62296023[C->A] | HG00683, HG01149, HG00689, HG00590 | HG00683, HG01149, HG00689, HG00590 | HG01173 |
| 11:62296084[T->G] | HG00651 | HG00651 | - |
| 11:62296190[C->G] | HG01108 | HG01108 | - |
| 11:62296535[T->C] | HG00651, HG00654, HG00334, HG01366, HG01790 | - | NA18603, HG01790, NA18572, HG00651, HG01137, HG00534, HG00699, HG00654, HG01375, HG00334, HG01366, HG00702, HG00613, HG00657, HG00689, HG00705 |
| 11:62296556[G->A] | HG01108 | - | - |
| 11:62296592[C->A] | HG01148, HG01134 | HG01134 | HG01148, HG00501 |
| 11:62296601[T->C] | HG00651, HG00654, HG00334, HG01366, HG01790, HG01108 | - | NA18603, HG00651, HG01125, HG00534, HG00699, HG00654, HG00334, HG01366, HG00613, HG00657, HG01503, HG01108, HG01506, NA18611, HG01173, HG01790, NA18572, HG00581, HG01137, HG00342, HG01375, HG00702, HG00328, HG00449, HG00689, HG00705 |
| 11:62296854[C->T] | HG01124 | HG01124 | - |
| 11:62296892[T->C] | HG00683 | HG00683 | - |
| 11:62296897[G->A] | HG00613 | HG00613 | - |
| 11:62296998[C->T] | HG01353 | HG01353 | - |
| 11:62297059[A->G] | NA18579 | NA18579 | HG00583, HG00334, HG00449, HG01350, HG00707, HG00607 |
| 11:62297162[G->A] | HG00651, HG00654, HG00334, HG01366, HG01790 | HG00334 | HG00651, NA18603, HG00534, HG00699, HG00654, HG01366, HG00702, HG00657, NA18572, HG01503, HG00705 |
| 11:62297320[A->G] | HG01108 | HG01108 | - |
| 11:62297410[A->G] | HG01108 | HG01108 | - |
| 11:62297447[T->C] | HG00651, HG00654, HG00334, HG01366, HG01790 | - | NA18603, HG01173, HG01790, NA18572, HG00651, HG01137, HG00534, HG00699, HG01375, HG00654, HG00334, HG01366, HG00702, HG00613, HG00657, HG00689, HG00705 |
| 11:62297462[A->G] | NA18609 | NA18609 | - |
| 11:62297759[T->C] | NA18563 | NA18563 | - |
| 11:62297789[C->T] | HG00583 | HG00583 | - |
| 11:62298074[C->G] | HG01108 | HG01108 | - |
| 11:62298597[C->A] | HG00463, HG00693, NA18603, HG00708, HG00699, HG00702, HG00657, HG00701, HG00689, HG00619, HG00308 | - | NA18603, HG00651, HG01500, HG01149, HG00693, HG00699, HG00534, HG00698, HG00654, HG00334, HG00614, HG00657, HG00613, HG00619, HG01503, HG01668, HG01107, HG01108, HG01506, NA18611, NA18537, NA18572, HG01170, HG00321, HG00683, HG01137, HG01374, HG01375, NA18609, HG00328, HG00449, HG00689, HG00607, HG00708, HG01125, HG00672, HG00671, HG01124, HG01790, HG01350, HG01791, HG01670, HG00581, HG00580, HG01390, HG01510, HG00464, HG00584, HG00463, HG00342, HG01512, HG00583, HG00263, HG01353, HG01354, HG00702, HG00701, HG00308 |
| 11:62298701[C->T] | HG01108 | HG01108 | - |
| 11:62298832[A->G] | HG01108 | HG01108 | - |
| 11:62299362[C->T] | NA18558 | NA18558 | - |
| 11:62299744[A->G] | HG01108 | HG01108 | - |
| 11:62299945[A->C] | HG00651, HG00654, HG00334, HG01366, HG01790, HG01108 | HG00334, HG01108 | HG00651, HG01125, NA18603, HG00534, HG00699, HG00654, HG01366, HG00702, HG00657, NA18572, HG00705, HG01506 |
| 11:62300276[C->G] | HG00684 | HG00684 | - |
| 11:62300502[G->A] | HG01108 | HG01108 | HG00663 |
| 11:62300711[C->T] | HG01108 | HG01108 | - |
| 11:62300713[C->T] | HG01108 | HG01108 | - |
| 11:62300953[C->T] | HG00464 | HG00464 | HG00702 |
